# The role of emotional labor in emotion regulation difficulties among social workers in Jordan

**DOI:** 10.3389/fpsyg.2026.1827645

**Published:** 2026-06-15

**Authors:** Rula Odeh Alsawalqa, Roqaya Alreesi, Ann Mousa Alnajdawi, Maissa N. Alrawashdeh, Ruba Saleh Alkam

**Affiliations:** 1 Department of Sociology, University of Khorfakkan, Khorfakkan, United Arab Emirates; 2 Department of Sociology, The University of Jordan, Amman, Jordan

**Keywords:** emotion regulation, emotional labor, Jordan, social work, social workers

## Abstract

Emotions represent essential resources in social work practice; however, difficulties in regulating them remain insufficiently explored, particularly in non-Western contexts. This study aimed to examine emotional labor (EL) strategies and difficulties in emotion regulation (DER) among social workers (SWs) in Jordan. A cross-sectional design was employed using a structured questionnaire administered to a purposive sample of 132 SWs in Amman. The findings indicated that SWs reported relatively low levels of EL strategy use and relatively high levels of DER, particularly in limited access to regulation strategies, impulse control difficulties, non-acceptance of emotional responses, and lack of emotional awareness. A statistically significant negative correlation was found between EL and DER (*r* = −0.330, *p* < 0.05), indicating that higher EL is associated with lower difficulties in emotion regulation. EL varied significantly by sex and educational level, with higher levels observed among females and those holding a master's degree. In contrast, DER differed significantly only by sex, with higher levels reported among males, and showed no significant variation across educational level or years of experience. These findings highlight the importance of strengthening emotional labor competencies as a potential mechanism for improving emotion regulation, professional effectiveness, and wellbeing among social workers. The study contributes to extending emotional labor research beyond Western contexts by providing context-specific evidence from Jordan and offering a more nuanced understanding of the relationship between EL and DER within socio-cultural and professional constraints.

## Introduction

Relationship-based social work is emotionally demanding, stressful, and complex. Social work is relatively dynamic, and social workers (SWs) bear a heavy emotional burden in their profession. They are involved in emotionally intense situations involving severe trauma and oppression, especially when dealing with those who have endured painful events such as seeking refuge and periods of heightened emotional stress such as poverty, death, violence, and hardship (Stanley and Bhuvaneswari, 2016; Howe, 2008; Ingram, 2013a; Ruiz-Fernández et al., 2021; Viviani, 2023). Therefore, they experience several mixed emotions, such as sadness, concern, guilt, anger, helplessness, anxiety, shock, humiliation, and agitation (Ruiz-Fernández et al., 2021; Stanley and Bhuvaneswari, 2016; Viviani, 2023; Morrison, 2006). They may also harbor fears regarding assault, personal safety, death, loss of control, separation from their organization, and relationships with colleagues or other professionals (Litvack et al., 2010; Smith et al., 2004).

Considering the challenging and complex nature of social work and the multiple demands and fears, an imbalance arises between what workers feel and the emotions required for their professional role (Viviani, 2023). Consequently, SWs are more likely to engage in high-risk behaviors and experience negative psychological and mental outcomes, including occupational distress, job burnout, depression, emotional exhaustion, isolation, fatigue, low emotional wellbeing, and emotional dissonance (Grant and Kinman, 2020; Ruiz-Fernández et al., 2021; Turtiainen et al., 2022; Viviani, 2023; Indregard et al., 2018). Therefore, SWs should be capable of emotional and cognitive regulation. They should have the emotional skills to respond appropriately to clients and empower them. They must identify and understand both their own emotional states and those of their clients to protect wellbeing, achieve high-quality services, maintain positive relationships, and enable goal-directed action (Turtiainen et al., 2022; Ingram, 2013a; Morrison, 2006).

Emotions are potent and constructive resources in social work practice and education (Dore, 2019; O'Connor, 2020) and are central to the experiences of SWs and clients (O'Connor, 2020). Emotions may be defined as “conscious mental reactions subjectively experienced as strong feelings usually directed toward a specific object and typically accompanied by physiological and behavioral changes” (Merriam-Webster, 2024; Gvelesiani et al., 2023). They serve as informational, motivational, and dynamic relational resources contributing to the evolution of consciousness (Izard, 2009; O'Connor, 2020) and manifest during SWs' core tasks, including client engagement, assessment, decision-making, collaboration, and dealing with stress (Morrison, 2006). Expressing or suppressing emotions is complex and difficult for SWs, especially given the diversity of clients' cultural, socioeconomic, and religious backgrounds. SWs' emotional job requirements are deeply rooted in and influenced by the sociocultural context and changes in welfare systems (Turtiainen et al., 2022). von Scheve (2012) indicated that emotion regulation is systematically shaped by culture and society, suggesting that the unequal distribution of emotional resources leads to discernible patterns in emotions and emotion regulation across different social groups.

Expressing emotions may sometimes be considered unprofessional, while suppressing or ignoring them can lead to difficulties for practitioners and clients. Therefore, emotional management and regulation are critical (Glumbíková et al., 2020; Ingram, 2013a). Separating emotions from practice is considered an anathema in interpersonal professions, as it disconnects the relational aspect from practice itself (Ingram, 2013a; Hennessey, 2011).

The emotional job requirements of SWs involve regulating their emotions to maintain neutrality and rational objectivity in problem identification and solution generation (Matthews, 2022). These requirements address emotional dissonance, the stress resulting from contradictions between felt and expressed emotions (Hochschild, 1983), which is associated with exhaustion, mental distress, and sickness absence (Indregard et al., 2018).

Emotional labor (EL) is highly prevalent in social work (Turtiainen et al., 2022). SWs may suppress emotions, but sometimes in ways that prevent them from leveraging emotions constructively in practice (Ferguson, 2005). Hochschild (1983) introduced EL as “the process by which workers are expected to manage their feelings in accordance with organizationally defined rules and guidelines” (Wharton, 2009). EL research addresses both organizational structures and the workers' efforts to express and regulate emotions and their consequences (Wharton, 2009). EL is “sold for wages,” giving it exchange value, and workers shape emotions according to “feeling rules” imposed by social structures (Alsawalqa et al., 2021; Gvelesiani et al., 2023). Hochschild (1979) highlighted that emotion management is interactive work to cope with feeling rules, which serve as ideological guides for emotional conduct.

Yang and Chen (2020) noted that empirical research on EL draws predominantly from emotion regulation theory, defined as “the processes by which people decide which emotions they have, when they have them, and how they experience and express these emotions.” Hochschild (1983) described two EL strategies: surface acting (displaying required emotions without changing actual feelings) and deep acting (modifying internal feelings to align with organizational rules). Ashforth and Humphrey (1993) argued these two strategies were insufficient, adding “expressing true feelings” as a main dimension (Diefendorff et al., 2005; Grandey, 2003; Kruml and Geddes, 2000; Yang et al., 2019).

SWs vary in focus and approach: some prioritize psychological aspects, others social, affecting their EL use (Buchbinder et al., 2004). Moesby-Jensen and Nielsen (2014) identified three types of EL in practice: shutting off emotions, deferring emotions, or succumbing to overwhelming emotions. EL may contribute to negative outcomes such as burnout and stress (Yikilmaz et al., 2024; Yang and Chen, 2020; Lee et al., 2019), or enhance socioaffective and sociocognitive processes (Salazar Kämpf et al., 2023). Constant emotional regulation can be draining, yet EL can also promote wellbeing, job satisfaction, productivity, and reduced personal problems when aligned with personality and job demands (Iplik et al., 2014; Johnson, 2004; Al Ma'aitah and Alsawalqa, 2021; Chu, 2002). Awareness of emotion regulation strategies improves service delivery and organizational practices (Aziz et al., 2018; Moesby-Jensen and Nielsen, 2014; Ingram, 2013a).

Professional credibility and legitimacy of the social work profession related to adherence to the rules of professional conduct, ethics, and values in practice are framed, shaped, and defined according to the nature of their work environments, national legal requirements, cultural and individual values, and backgrounds of clients and SWs themselves (Alnajdawi and Alsawalqa, 2024; Malinga et al., 2018). EL is related to a certain emotional culture and the community in the workplace, where the regulation and expression of the feelings of SWs are managed in professional and organizational contexts, along with personal contexts (Moesby-Jensen and Nielsen, 2014; Winter et al., 2019). These relationships may result in performance discrepancies, occupational challenges, and difficult cognitive and emotional regulation by SWs. They may also lead to ethical dilemmas that are directly connected to the value base of practice. Ethical dilemmas may force SWs to take professional and emotional decisions that may conflict with ethical principles, institutional procedures, and country law, leading to professional problems and client dissatisfaction (Alnajdawi and Alsawalqa, 2024; Kadushin and Egan, 2001; Reamer, 2019).

In Jordan, social work as a profession is not completely acknowledged at the state policy level. There is no defined job title at the Civil Service Commission for recruiting social work graduates, and the present code of ethics cannot be considered a de facto guide for professional behavior. This does not help SWs to practice professionally. Additionally, the lack of fiscal incentives and human resources, clash between the ethical principle of self-determination and the cultural norms of clients, and lack of cross-team collaboration all contribute to multiple ethical dilemmas for Jordanian SWs. This leads to poor job performance and quality of services, weakening objectivity in professional relationships, and experiencing colleague and client-related negative emotions (Alnajdawi and Alsawalqa, 2024). Moreover, Alnajdawi et al. (2025) found that social workers in Jordan experience job burnout and workplace bullying, along with low levels of social capital.

Given the relative novelty of the social work profession in Jordan, the shortage of specialized professionals, lack of a solid knowledge base, and ethical dilemmas, Jordanian SWs strongly need to perform EL. This is especially important in the sociocultural context that does not recognize the nature and benefits of the social work profession, which consequently receives inadequate attention. This context, which is still patriarchal and tribal despite its civilization, elicits certain emotions driven by a complex mixture of Arab and religious norms and traditions, which affects social life, professional life, and the fulfillment of emotional demands (Alnajdawi and Alsawalqa, 2024). Furthermore, Jordanian researchers have neglected to address issues related to the psychological and mental health of SWs to improve their performance.

Despite the growing body of research on emotional labor (EL) and emotion regulation, several critical gaps remain. First, the majority of empirical studies have been conducted in Western contexts, with very limited attention to Arab settings, particularly Jordan, where socio-cultural norms, gender roles, and the professional status of social work differ substantially. Second, although emotional labor has been widely examined in relation to burnout and wellbeing, its relationship with difficulties in emotion regulation (DER) remains insufficiently explored, especially within social work practice. Third, prior research has rarely integrated emotional labor theory with established models of emotion regulation to provide a comprehensive understanding of how emotional strategies function not only as job demands but also as regulatory capacities.

Accordingly, this study offers several novel contributions. It is among the first to empirically investigate the relationship between emotional labor (EL) and difficulties in emotion regulation (DER) among social workers in Jordan. It further integrates Hochschild's emotional labor framework with Gratz and Roemer's model of emotion regulation within a non-Western socio-cultural and professional context. Importantly, the study provides evidence suggesting that emotional labor may function as a context-dependent regulatory resource, potentially reducing difficulties in emotion regulation, rather than solely acting as a source of strain. By doing so, this research extends existing theoretical perspectives and contributes to a more context-sensitive and globally inclusive understanding of emotional processes in social work practice.

Therefore, in response to these gaps, the current study aimed to investigate emotional labor (EL) strategies among Jordanian social workers and the associated difficulties in emotion regulation (DER) within professional practice. Specifically, the study examined: (1) the levels of EL strategies, (2) the levels of difficulties in emotion regulation, (3) the association between EL and DER, and (4) differences in EL and DER according to sex, educational level, and years of experience.

### Theoretical and conceptual framework

Based on Hochschild's EL theory and Gratz and Roemer's DER model, the Jordanian socio-cultural and professional context is expected to shape specific patterns of EL strategy use and difficulties in emotion regulation among social workers. Socio-cultural and professional factors such as patriarchal norms, tribal affiliations, limited state recognition of social work, organizational constraints, and culturally defined gender expectations may act as moderators influencing how social workers implement EL strategies and manage emotional difficulties. For instance, female SWs may rely more on surface acting due to societal expectations, while male SWs may face greater challenges regulating naturally felt emotions. Investigating EL and DER among Jordanian SWs allows us to explore how these theoretical frameworks apply in a context characterized by these socio-cultural and professional conditions.

Therefore, the current study aimed to explore several key aspects of EL and difficulties in emotion regulation among Jordanian SWs. First, it sought to examine the overall levels of EL strategies and the difficulties experienced during emotion regulation in professional practice. Second, the study aimed to investigate the association between EL strategies and difficulties in emotion regulation, in order to understand how the use of emotional labor might relate to reduced or increased challenges in managing emotions. Third, the study intended to identify potential differences in EL strategy use and emotion regulation difficulties according to demographic factors, including sex, educational level, and years of experience. Finally, the study aimed to explore the specific EL strategies most frequently employed by male and female SWs, as well as the main challenges they face in regulating their emotions during professional practice. Understanding how Jordanian social workers navigate emotional labor and emotion regulation difficulties within their unique socio-cultural and professional context provides critical insights for enhancing social work education, training, and policy development in the Arab world.

**Figure 1:**
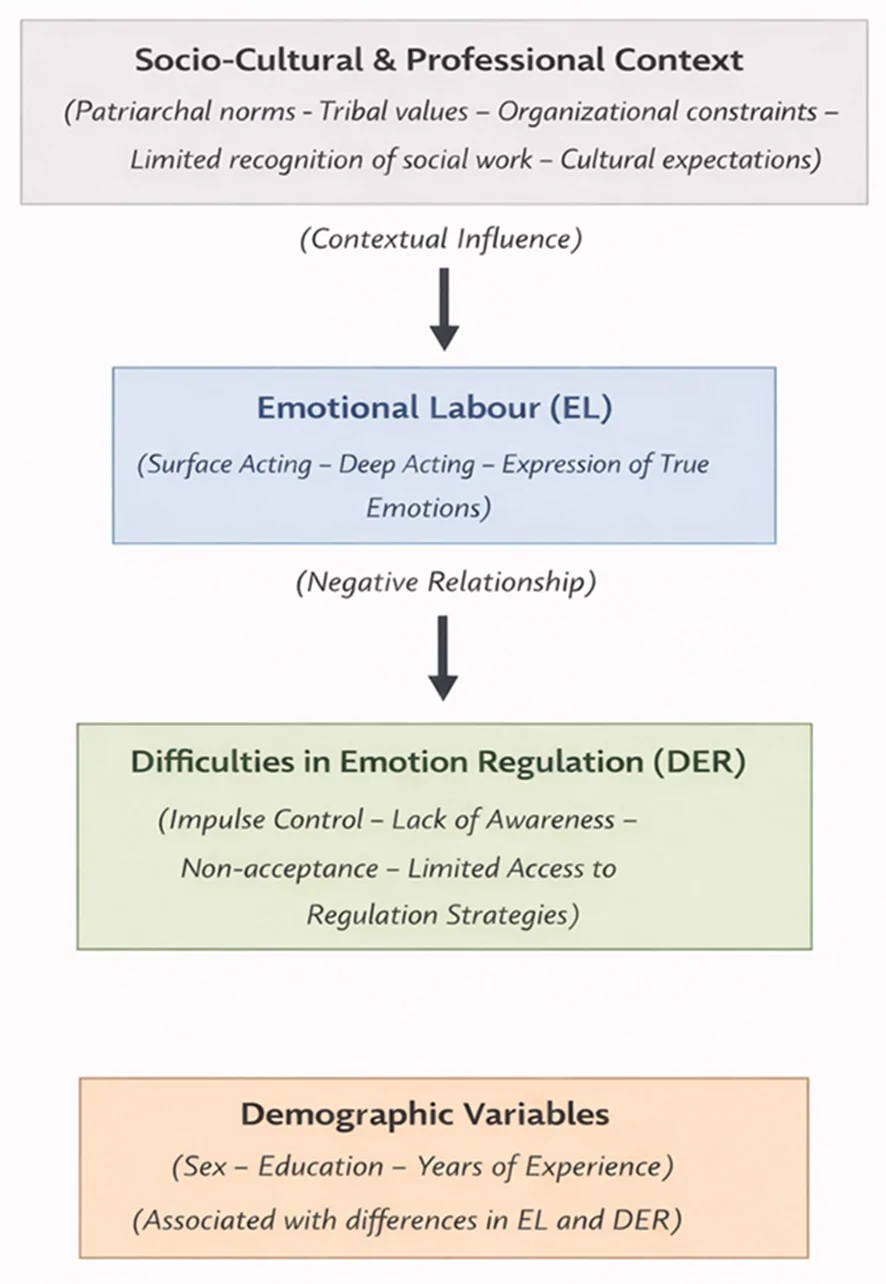
Conceptual framework of the study.

Based on Hochschild's emotional labor theory and Gratz and Roemer's model of difficulties in emotion regulation, the present study conceptualizes emotional labor (EL) as a regulatory process that may influence the level of difficulties in emotion regulation (DER). Specifically, EL strategies (i.e., surface acting, deep acting, and expression of naturally felt emotions) are expected to be associated with DER levels, as they reflect individuals' capacity to manage emotional experiences in professional contexts. In this framework, EL is treated as the main explanatory variable, whereas DER represents the outcome variable. Sociodemographic variables (sex, educational level, and years of experience) are not modeled as predictors but are examined to identify potential group differences in EL and DER levels. As shown in Figure 1, the conceptual framework illustrates the direct association between EL and DER, alongside the role of sociodemographic variables in explaining variations between groups.

## Methods

A purposive sampling strategy was employed to recruit participants who were most relevant to the research objectives. The included participants held bachelor's and/or master's degrees in social work, including individuals qualified through professional training courses. Given that the social work profession in Jordan has only recently gained serious attention and no official statistics exist regarding the total number of social workers, purposive sampling was considered appropriate to ensure that participants possessed sufficient knowledge, experience, and active engagement in social work practice.

Focusing on Amman allowed access to a diverse pool of practitioners and trainees from multiple institutions and service settings, thereby providing a preliminary yet contextually grounded understanding of emotional labor (EL) and difficulties in emotion regulation (DER) within the Jordanian context. Data were collected between 28 December 2024 and 18 March 2025. Participants completed an online questionnaire accessed through an anonymous link distributed via social media platforms such as WhatsApp and email. The study objectives were clearly explained within the questionnaire, along with an informed consent item to confirm voluntary participation. Sociodemographic characteristics and study measures were also included. Additionally, 16 questionnaires were manually distributed upon the request of some participants.

The final sample comprised 132 participants. While the study does not aim for statistical generalization due to the use of purposive sampling and the absence of reliable population statistics, the adequacy of this sample size can be supported from a statistical perspective. In correlational research, sample size is closely related to the magnitude of the effect size rather than a fixed numerical threshold. According to Cohen (1988), a correlation coefficient of approximately 0.30 represents a medium effect size, which can typically be detected using moderate sample sizes. The observed relationship in this study (*r* = −0.330) falls within this range, indicating that the sample size was sufficient to detect a meaningful and statistically interpretable association between emotional labor and difficulties in emotion regulation. Furthermore, the sample size is adequate for the statistical techniques employed in this study, including Pearson correlation, independent-samples *t*-tests, and one-way ANOVA, which are commonly applied in social science research with comparable sample sizes. Thus, the selected sample provides a reasonable balance between feasibility, contextual relevance, and statistical validity.

### Measures

#### The emotional labor strategies (ELs) score

This scale was developed by Diefendorff et al. (2005) to measure three types of EL strategies: surface acting (seven items), deep acting (four items), and the expression of naturally felt emotions (three items). Each item is rated on a five-point Likert scale ranging from one (strongly disagree) to five (strongly agree).

#### The DERS score

This scale was developed by Gratz and Roemer (2004) and measures emotion regulation problems. The 36-item self-reported questionnaire is used to determine how respondents relate to their emotions through a Likert scale of one (almost never) to five (almost always) and the following six domains: (1) impulse control difficulties (six items), (2) limited access to emotional regulation strategies (eight items), (3) non-acceptance of emotional responses (six items), (4) difficulties engaging in goal-directed behavior (five items), (5) lack of emotional awareness (six items), and (6) lack of emotional clarity (five items).

### Statistical analysis

This study used IBM SPSS Statistics (version 25; IBM Corp., Armonk, NY, USA) for the data analyses, including descriptive and inferential statistics. Cronbach's alpha (α) was used to estimate the internal consistency of measures. The Kolmogorov–Smirnov test was used to test the normality of the distribution of data with a degree of freedom parameter. The Split-Half Reliability (S-HR) was used to verify construct validity. Additionally, multivariate analysis of variance was used to simultaneously examine the effects of multiple independent variables on the dependent variables. Pearson's correlation coefficient (*r*) was used to quantify the association between EL and DER and explore the direction of the linear relationship between them.

## Results

The final sample comprised 132 social workers (SWs) from diverse organizations and occupations in Amman, the capital of Jordan. Most participants were female (66.7%) and aged between 25 and 35 years. In terms of education, 68.2% had completed a B.A. degree and 31.8% held an M.A. degree. Regarding work experience, 59.8% had 1–5 years of experience.

The measures demonstrated satisfactory internal consistency reliability, with Cronbach's α values of 0.885 for EL and 0.891 for DERS. The data met normality assumptions, with skewness and kurtosis values of −0.302 and 0.696 for EL and −0.138 and 0.639 for DERS. The S-HR analysis indicated good content validity (EL, S-HR = 0.909; DERS, S-HR = 0.888), exceeding the recommended threshold of 0.70 (Sekaran and Bougie, 2016). Overall, Table 1 shows that SWs reported relatively low ability to apply EL strategies and relatively high levels of difficulties in emotion regulation.

**Table 1:** Means and standard deviations of the levels of implementation of emotional labor strategies and levels of the difficulties in emotion regulation among Jordanian social workers.

Factors	*M*	SD
Emotional labor	1.91	0.73
Surface acting	2.16	1.37
Deep acting	1.78	1.16
Expression of naturally felt emotions	1.78	1.09
Difficulties in emotion regulation	3.94	0.85
Non-acceptance of emotional responses	3.98	0.90
Difficulty engaging in goal-directed behavior	3.83	1.08
Impulse control difficulties	4.09	0.77
Lack of emotional awareness	3.90	1.00
Limited access to emotion regulation strategies	4.00	0.85
Lack of emotional clarity	3.84	1.01

The Pearson correlation coefficients (Table 2) revealed a negative association between EL and DER (***r*** **= −0.330**), indicating that higher levels of EL were linked to lower levels of DER. Independent-samples *t*-tests and one-way ANOVA (Tables 3, 4) showed significant differences in EL according to sex (favoring females) and educational level (favoring those with a master's degree) (*p* < 0.001). Similarly, DER levels differed significantly by sex (favoring males) (*p* < 0.001). No significant differences were observed for DER according to educational level (*p* = 0.290). No significant differences were observed for years of experience in either EL (*p* = 0.123) or DER (*p* = 0.110).

**Table 2:** Pearson correlation analysis of emotional labor and difficulties in emotion regulation among Jordanian social workers.

Variables	Correlation (R)	Emotional labor
Difficulties in emotion regulation	(*R*)	−0.330^**^
	*Sig*	0.00

^*^Correlation is significant at the 0.01 level (two-tailed).

**Table 3:** Means and standard deviations of the indexes for the attitudes toward emotional labor and difficulties in emotion regulation due to the independent variable.

Variables		No.	Emotional labor		Difficulties in emotion regulation	
			*M*	SD	*M*	SD
Sex	Female	88	2.07	0.78	3.62	0.96
	Male	44	1.84	0.65	4.10	0.36
Educational level	B.A.	90	1.78	0.63	3.79	0.85
	M.A.	42	2.20	0.76	4.27	0.72
Years of experience	1–4 years	79	1.94	0.72	3.82	0.88
	5–10 years	53	1.88	0.67	4.12	0.74

**Table 4:** Multivariate analysis of variance results.

Source	Emotional labor				Emotion regulation difficulties			
	Sum of Squares	df	F	Sig	Sum of Squares	df	F	Sig
Sex	5.321	1	13.841	0.000	8.412	1	13.986	0.000
Educational level	10.138	1	26.374	0.000	2.931	1	4.872	0.290
Years of experience	0.926	1	2.409	0.123	1.559	1	2.592	0.110
Error	47.667	124			74.579	124		
Total	550.723	132			2,147.186	132		

Descriptive statistics indicated that females more frequently used surface acting strategies to regulate emotions, whereas males more often relied on the expression of naturally felt emotion. The main areas of difficulty in emotion regulation for both sexes included limited access to emotion regulation strategies, impulse control difficulties, non-acceptance of emotional responses, and lack of emotional awareness.

## Discussion

The social work profession is an occupation that requires emotional labor (EL). Social workers (SWs) are expected to regulate their emotions to prevent harmful psychological, emotional, and mental consequences resulting from heavy emotional job requirements. Emotional regulation and its associated difficulties in social work practice remain largely unexamined, particularly in the Jordanian context. To our knowledge, this study is one of the first to address EL strategies and explore difficulties in emotion regulation (DER) among SWs in Jordan. The purpose of this study was to contribute positively to the literature on the role of emotions in social work practice by revealing the levels of SWs' abilities to practice EL strategies, types of emotion regulation in professional practice, and difficulties in emotion regulation. In addition, we aimed to understand the role of personality traits and sociodemographic factors in predicting these levels.

Our results revealed that SWs have limited abilities to practice EL strategies and experience marked difficulty in regulating their emotions, including limited access to emotion regulation strategies, impulse control difficulties, non-acceptance of emotional responses, and lack of emotional awareness. Moreover, increasing the ability to practice these strategies in professional practice can reduce the rates of emotion regulation difficulties. Interestingly, our findings indicated that higher EL was associated with lower DER (a negative correlation). While this appears counterintuitive relative to literature linking EL to burnout, it may suggest that successfully performing EL strategies acts as a protective mechanism in the Jordanian socio-cultural and professional context. In other words, SWs who are competent in practicing EL may be better equipped to manage emotional challenges, reducing difficulties in emotion regulation. This protective effect likely interacts with cultural norms, gender expectations, and professional constraints, illustrating the value of integrating socio-cultural moderators into the EL-DER framework.

This finding can be further interpreted in light of the emotionally demanding nature of social work, as outlined in the introduction. Social workers operate in highly stressful environments characterized by emotional intensity, exposure to trauma, and constant interpersonal demands, which require continuous emotional regulation. In such contexts, emotional labor may function not only as a job requirement but also as an adaptive regulatory mechanism (Grandey, 2000; Gross, 1998).

From an emotion regulation perspective, engaging in emotional labor strategies such as surface acting, deep acting, and the expression of naturally felt emotions may enhance individuals' capacity to monitor, adjust, and manage their emotional responses in demanding professional situations. Given the high levels of emotional dissonance and psychological strain associated with social work, those who are more capable of performing emotional labor may develop more effective regulatory skills over time. This may explain why higher levels of emotional labor are associated with lower difficulties in emotion regulation in the present study.

Furthermore, this relationship may be reinforced by the Jordanian socio-cultural and professional context, where emotional expression is shaped by strong cultural norms, gender expectations, and limited professional recognition. In such environments, social workers may rely more heavily on emotional labor as a practical tool to navigate complex emotional and social demands. Repeated engagement in these strategies may contribute to increased emotional awareness and improved regulatory capacity, thereby reducing difficulties in emotion regulation.

This interpretation helps reconcile the current findings with previous literature that has predominantly emphasized the negative consequences of emotional labor, suggesting that its impact may vary depending on contextual, cultural, and professional conditions, as well as the level of individual competence in applying emotional labor strategies.

EL is the dynamic integration of emotional requirements, emotion regulation, and emotion performance (Grandey and Gabriel, 2015), which can reduce discrepancies between emotional expressions and organizationally defined rules using emotion regulation strategies (Diefendorff and Gosserand, 2003). Emotion regulation training may foster empathy and compassion and alleviate empathic distress (Salazar Kämpf et al., 2023). Emotion regulation strongly correlates with social communication (Martínez-González et al., 2022), and the capacity to use relationships to address the needs of service users involves social work competence, which includes effectively handling both personal emotions and those of others (Morrison, 2006). The ability to manage personal emotional reactions efficiently is central to the role of SWs, especially in complex care settings (Herland, 2022). Employees who display emotions are likely to appear sincere and provide good service (Ashforth and Humphrey, 1993). Chu (2002) theoretically predicts that individuals with strong empathy are more likely to perform emotional labor in a more genuine manner and experience fewer negative consequences.

Emotional dysregulation refers to the inability to control or regulate emotional responses using healthy strategies. Positive emotions amidst negative situations play an important role in the psychological resilience and coping of SWs (Collins, 2007). Individuals who regularly experience intense negative emotions are more likely to rely on unhealthy strategies such as self-harm (Rolston and Lloyd-Richardson, 2022). Difficulties in regulating emotions refer to the inability to be aware of, understand, and accept emotions; the inability to control impulsive behaviors and act according to desired goals when faced with negative emotions; and the difficulty in accessing effective strategies for regulating emotions (Gratz and Roemer, 2004). Alrawashdeh et al. (2024) revealed that employees who engage in detached engagement demonstrate impulse control, anticipate emotions less, exhibit less emotional countenance, and have high levels of negative emotion regulation; these employees are more likely to experience difficulties with emotion regulation. SWs need to be able to hear, understand, and regulate the perspectives and emotions of clients to establish and maintain trust (Ingram, 2013a, 2015).

Situational and individual factors influence EL performance, including strategies and how others receive and respond to these strategies. These factors include seniority, institution type, task routineness, form of interaction, job autonomy, and job satisfaction, which affect self-presentation and emotion regulation. Controlling expressive behavior to suit interactive situations and reducing self-censorship to maintain true internal feelings contributes to reducing tension and emotional distraction (Chu, 2002; Mahamad, 2018). Emotional intelligence components, especially emotional clarity and emotional repair (Chinchilla et al., 2024), are positively associated with SWs' role efficacy (Singh and Sharma, 2006) and contribute to developing the social work/service user relationship. These components can be integrated with empathy and emotion regulation into a model for practice (Ingram, 2013b). Emotion management, emotion regulation, and emotional intelligence are concepts central to processes involved in emotion modification. Measures of emotional intelligence often appraise individuals' ability to regulate mood and manage emotions (Abdul Hamid, 2023; Mayer et al., 2008). Eggli et al. (2022) found that EL strategies and disengaging work styles may have beneficial effects on SWs at both day-to-day and intra-individual levels.

The higher use of surface acting among female SWs aligns with societal expectations in Jordan's patriarchal and tribal context, where women may be expected to display socially appropriate emotions while suppressing personal feelings. Conversely, male SWs more frequently expressed naturally felt emotions but faced greater difficulties in regulating them, reflecting different social pressures and cultural norms regarding emotional expression for men. Educational level and years of experience also interact with these socio-cultural moderators, shaping both EL strategy adoption and the ability to manage emotional difficulties. This demonstrates how the proposed framework can interpret variations in EL and DER within the Jordanian socio-cultural and professional context.

Regarding sociodemographic variables, our results found that female SWs had a slightly higher ability than males in performing EL strategies during professional practice and tended to use surface acting. Males, on the other hand, tended to express naturally felt emotions and experienced slightly higher levels of difficulty regulating their emotions. Educational level significantly predicted EL, but showed no significant association with DER, whereas years of experience showed no significant influence on either variable, whereas years of experience showed no significant influence. These findings align with prior literature (Viviani, 2023; Al Ma'aitah and Alsawalqa, 2021; Abdul Hamid, 2023). Intense social stressors and organizational factors may also predict more intensive surface acting, deep acting, and post-work disengagement (Yikilmaz et al., 2024).

Sex affects both the frequency and type of EL experienced, including strategies and reception by others (Mahamad, 2018; Rasheed-Karim, 2020). Gendered expectations impose differing emotion regulation rules and expose culturally available strategies (Abdul Hamid, 2023). Evidence regarding differences in EL and DER by sex is mixed: some studies show females exhibit more intensive EL and use surface acting (Al Ma'aitah and Alsawalqa, 2021; Babalola and Nwanzu, 2022), whereas others show higher EL through deep acting or more surface acting among males (Akin et al., 2014; Yilmaz et al., 2015). In Jordan, emotional culture and social positioning influence these patterns (Alsawalqa et al., 2021).

Effective emotion regulation allows SWs to engage less in self-injurious behaviors, accept feelings arising from professional or social pressures, make intentional and appropriate decisions, enhance wellbeing, and improve professional and personal relationships (Carminati, 2021; Côté et al., 2010; Ford and Feinberg, 2020; Martínez-González et al., 2022). Emotion regulation training may foster empathy, compassion, and alleviate empathic distress (Salazar Kämpf et al., 2023). Experience also plays a role in emotion regulation (Ruiz-Fernández et al., 2021; Abdul Hamid, 2023). Most SWs in this study had limited experience and practiced social work with unclear professional foundations, ethical dilemmas, and in a society with limited recognition of the profession (Alnajdawi and Alsawalqa, 2024).

These findings reconfirm that social work practice involves interactive work and emotional assessment (Turtiainen et al., 2022). Educating SWs on strategies to regulate their emotions is crucial to maintain psychological wellbeing, enhance service quality, and inform supervisors and policymakers about potential dilemmas in practice. Future research should further investigate EL strategies, personal traits influencing emotional regulation, and develop evidence-based interventions, particularly for male SWs and those facing high DER levels.

By explicitly integrating socio-cultural and professional moderators of the Jordanian context into the study of emotional labor (EL) and difficulties in emotion regulation (DER), this research extends existing EL and DER theories beyond Western settings. It demonstrates how cultural norms, gender roles, organizational factors, and professional recognition shape emotional regulation processes. This approach enriches theoretical understanding, highlights the importance of contextual variables, and provides a foundation for future research in non-Western contexts. Consequently, the study contributes to a more globally inclusive perspective on emotional labor in social work, showing that theoretical models must account for local socio-cultural and professional conditions to accurately explain emotion regulation patterns.

### Limitations

This study has several limitations that should be considered when interpreting the findings. First, the sample was limited to social workers in Amman, Jordan, which may restrict the generalizability of the results to other regions or countries. *While focusing on Amman enabled access to a diverse pool of practitioners and trainees from multiple institutions and service settings, this sampling strategy may limit the representativeness of social workers in rural areas or other regions of Jordan*. Second, the cross-sectional design prevents any causal inferences regarding the relationships between emotion regulation strategies and associated difficulties. Third, the data were collected using self-reported measures, which may be subject to social desirability bias and inaccuracies in recall. Finally, other potentially relevant variables, such as organizational culture or workload, were not examined and may have influenced the results. Future research employing longitudinal or mixed-method designs and more diverse samples is recommended to provide a deeper understanding of these associations. Given these limitations, the findings regarding sex and educational differences in EL and DER should be interpreted with caution.

## Conclusion

Given the importance of emotions and power relationships in social work practice (Herland, 2022), professional social workers (SWs) actively shape emotional interactions through emotional labor (EL) strategies as part of their interventions (Schröder, 2020). They frequently encounter complex situations involving trauma, suffering, violence, abuse, and vulnerability, which may trigger intense emotional responses (Douglas, 2013; Horwitz, 1998) and adversely affect their wellbeing, performance, and service quality if not effectively managed.

The findings of this study indicated that SWs reported relatively low levels of EL and high levels of difficulties in emotion regulation (DER), particularly in limited access to regulation strategies, impulse control difficulties, non-acceptance of emotional responses, and lack of emotional awareness. Female participants reported higher levels of EL, whereas males reported higher levels of DER. In addition, females tended to use surface acting more frequently, while males more often relied on the expression of naturally felt emotions.

Furthermore, EL was significantly associated with both sex and educational level, with higher levels observed among females and those holding a master's degree. In contrast, DER differed significantly only by sex and showed no significant variation across educational level or years of experience. A negative association between EL and DER suggests that higher levels of EL are linked to lower difficulties in emotion regulation.

These findings underscore the importance of enhancing emotional labor competencies among SWs as a potential pathway to improving emotion regulation, professional effectiveness, and overall wellbeing. Policymakers in the Jordanian social development sector are encouraged to support targeted training and capacity-building initiatives that strengthen emotional regulation skills, with expected benefits for both individual practitioners and organizational practices.

## Data Availability

The original contributions presented in the study are included in the article/supplementary material, further inquiries can be directed to the corresponding author.
